# Risk of cancer following presentation with new-onset atrial fibrillation using data from UK national databases

**DOI:** 10.1093/europace/euaf319

**Published:** 2025-12-11

**Authors:** Hiroyuki Yoshimura, Nadine Zakkak, Georgios Lyratzopoulos, Gregory Y H Lip, Floriaan Schmidt, Rui Providencia

**Affiliations:** Institute of Health Informatics Research, University College London, 222 Euston Road, London NW1 2DA, UK; Epidemiology of Cancer Healthcare and Outcomes Group (ECHO), Department of Behavioural Science and Health, Institute of Epidemiology and Health Care, University College London, London, UK; Cancer Intelligence, Cancer Research UK, London, UK; Epidemiology of Cancer Healthcare and Outcomes Group (ECHO), Department of Behavioural Science and Health, Institute of Epidemiology and Health Care, University College London, London, UK; Liverpool Centre for Cardiovascular Science at University of Liverpool, Liverpool John Moores University and Liverpool Heart & Chest Hospital, Liverpool, UK; Danish Center for Health Services Research, Department of Clinical Medicine, Aalborg University, Aalborg, Denmark; Department of Cardiology, Lipidology and Internal Medicine with Intensive Coronary Care Unit, Medical University of Bialystok, Bialystok, Poland; Institute of Cardiovascular Science, University College London, London, UK; Department of Cardiology, Division Heart and Lungs, University Medical Center Utrecht, Utrecht, The Netherlands; Institute of Health Informatics Research, University College London, 222 Euston Road, London NW1 2DA, UK; Barts Heart Centre, Barts Health NHS Trust, London, UK

**Keywords:** Arrhythmia, Neoplasia, Prediction, Prognosis, Comorbidity

## Abstract

**Aims:**

Atrial fibrillation (AF) and cancer are both highly prevalent conditions and are known to be associated. Our aim was to identify predictors and develop models for all cancer types, in men and women, and for the four most common cancer types in the AF population using linked primary and secondary care data from the UK.

**Methods and results:**

We included 163 549 patients diagnosed with AF between January 1998 and May 2016, and no previous history of cancer. Following TRIPOD methodology, we developed a ridge-penalized multivariable logistic regression model to predict 1-year cancer incidence after AF diagnosis, using 70% of the data for derivation and 30% for validation. Age was associated with an increased risk across all cancer types. Socioeconomic deprivation, smoking, excessive alcohol intake, family history of cancer, chronic kidney disease, anaemia, and several cancer-related symptoms and clinical signs (e.g. rectal bleeding, loss of appetite) were associated with an increased risk in one or more cancer types. The prediction models showed moderate-to-good discrimination in the validation set, with c-statistic of 0.69 (0.68–0.70) for all cancer in men, 0.63 (0.62–0.65) for all cancer in women, 0.70 (0.68–0.73) for lung cancer, 0.70 (0.66–0.73) for colorectal cancer, 0.59 (0.53–0.65) for breast cancer, and 0.78 (0.72–0.84) for prostate cancer.

**Conclusion:**

Most of the identified potential risk factors for cancer in the AF population are also associated with cardiovascular disease. The 1-year cancer prediction models showed moderate to good predictive performance and may help improve the management of patients with AF.

## Graphical Abstract

**Graphical Abstract:**
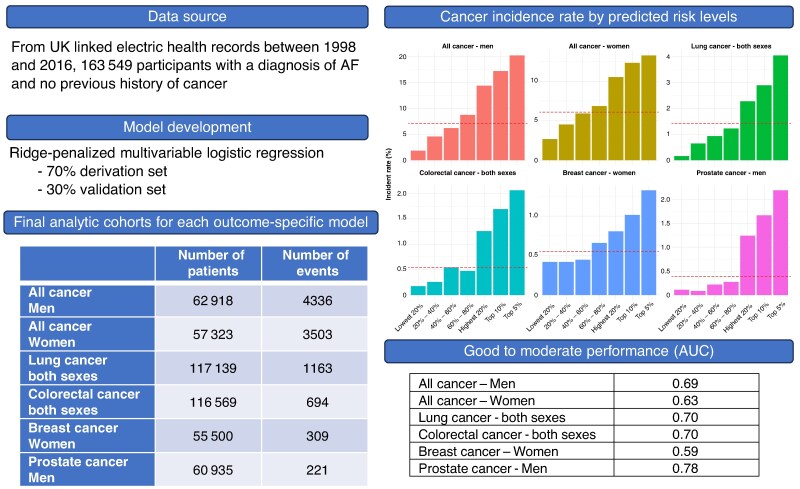

What’s New?

- Using data from 163 549 patients with a diagnosis of AF and no previous history of cancer in UK national databases, 1-year cancer prediction models achieved moderate-to-good performance (AUC 0.59–0.78)
- The cancer risk prediction model for patients with new-onset AF may support risk stratification and guide integrated AF management, but external validation in independent cohorts is warranted.

## Introduction

Atrial fibrillation (AF) is one of the most common cardiovascular conditions. The prevalence of AF has more than doubled over the past 30 years, with prevalence according to the Global Burden of Disease Study 2021 standing at 59.7 million, and further increases are expected due to an ageing population.^1,2^ Similarly, cancer remains a major global health burden. The International Agency for Research on Cancer estimated that there were nearly 20 million new cases of cancer and 9.7 million cancer-related deaths in 2020.^3^ Lung cancer, female breast, colorectal, and prostate cancer are the most common cancer types, accounting for nearly 41% of all diagnoses worldwide,^3^ and over half of new cancer diagnoses in the UK.^4^ Bray *et al.*^3^ predict that by 2050, annual cancer cases will reach 35 million, underscoring the urgency of investing in prevention and targeting modifiable risk factors globally.

A growing body of evidence has reported bidirectional associations between AF and cancer.

Cancer is associated with an increased risk of AF, and AF is also linked to a higher incidence of cancer.^5–8^ The prevalence of coexisting AF and cancer has been increasing and is associated with higher all-cause mortality. Cancer is observed in ∼13% of patients with AF—nearly three times the prevalence seen in individuals without AF.^5^ A large UK cohort study suggests that AF may be a paraneoplastic manifestation of occult cancer, due to an increase in new cancer diagnoses in the early period after the onset of AF.^8^

When compared with ischaemic heart disease and heart failure, a growing body of evidence shows that AF exhibits distinct pathophysiological mechanisms leading to an AF-specific signature in the disease-related complications.^9^ For example, with regards to stroke, AF patients possess a different thrombo-embolic profile—predominantly cardioembolic rather than atherothrombotic or hypoperfusion-related. When considering processes that may also play a role in cancer, inflammation in AF is often localized to atrial tissue, and the patterns of neurohormonal activation and oxidative stress differ from those seen in other cardiovascular diseases.^10^

Patients with AF have complex comorbidity patterns and experience different clinical trajectories from the general population.^11^ For instance, models designed to estimate the risk of stroke and systemic embolism in the general population frequently demonstrate diminished predictive accuracy when applied to individuals with AF.^12^ Hence, risk prediction models for early detection and personalized management of frequent comorbid conditions in the AF populations are warranted. Early identification of AF patients at high risk of incident cancer is crucial for enabling timely screening and tailored therapy, which can lead to better outcomes and long-term survival. To the best of our knowledge, no previous study has assessed the potential risk factors for incident cancer in the AF population, or identified AF patient subgroups at higher risk of cancer diagnosis.^3,13^

Therefore, using linked UK electronic health records, we aimed to develop prediction models for the 1-year risk of the four most common cancer types and all cancers in individuals with AF.

## Methods

### Data sources

We used linked, nationwide electronic health records from the UK, combining three datasets: Primary care records from the Clinical Practice Research Datalink (CPRD), secondary care records from the Hospital Episodes Statistics (HES), and death registration data from the Office for National Statistics (ONS). The CPRD includes information from UK primary care practices on demographics, diagnoses, symptoms, drug prescriptions, vaccinations, laboratory tests, and referrals to hospital or specialist care, covering ∼2.9 million current patients (around 4.3% of the UK population).^14^ The HES contains clinical information on patients’ hospital activity and treatment. The HES includes clinical information on hospital admissions, diagnoses, and treatments.

#### Features (exposures)

READ codes and International Classification of Diseases, Tenth Revision (ICD-10) codes from the Health Data Research UK (HDR-UK) Phenotype Library were utilized for identifying comorbidities and outcomes.^15^ In the UK, an electrocardiogram tracing is required for making a diagnosis of AF.^16^

We examined potential risk factors associated with AF and cancer. Potential risk factors for AF were selected based on those commonly observed in the AF population.^17^ To identify factors associated with cancer risk, we included variables reported in the QCancer algorithms, which estimate the absolute risk of an existing, undiagnosed cancer in the general population based on combinations of symptoms and readily available clinical risk factors.^18,19^ Cancer-specific variables, such as symptoms characteristic of each cancer type, were included only in the corresponding cancer prediction model. Previously published systematic reviews suggest that, among patients later diagnosed with cancer, changes in healthcare use, and time from first symptoms to cancer diagnosis is usually months.^20,21^ Therefore, as our risk schemes are based on the hypothesis that AF in some patients behaves as a paraneoplastic syndrome, with some patients having undiagnosed cancer when they develop new-onset AF, the variables related to current symptoms or recent occurrence were collected based on events that occurred within 1 year before AF diagnosis.^20^ Therefore, variables related to current symptoms or recent occurrence were collected based on events that occurred within 1 year before AF diagnosis. Socioeconomic deprivation was assessed using the English Indices of Deprivation 2015 and categorized into quintiles. Anaemia was defined using both diagnostic codes and haemoglobin levels (below 130 g/L for men, and below 120 g/L for women). For both C-reactive protein (CRP) and prostate-specific antigen (PSA), many participants had no test results available. Therefore, we created a separate category for ‘not tested’ and classified these variables into three categories: ‘not tested’, ‘normal’, and ‘elevated’. Based on previous publications, elevated CRP (with a cut-off of ≥10 mg/L) was also examined as a potential predictor.^22^ For PSA, elevation was defined using age-specific thresholds: 2.5 ng/mL for men aged <50 years, 3.5 ng/mL for 50–59 years, 4.5 ng/mL for 60–69 years, and 6.5 ng/mL for ≥70 years.^23^ All codes used are listed in Supplementary material online, *Table S1*.

### Study sample

**Figure 1:**
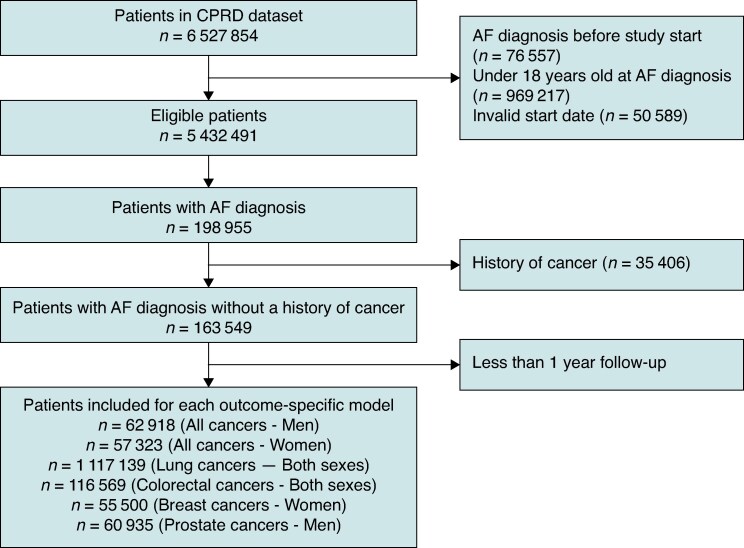
Study flow chart. AF, atrial fibrillation; CPRD, Clinical Practice Research Datalink.

*Figure 1* shows the flowchart of patient selection. After excluding patients in CPRD who were not linked to HES or were under 18 years of age, a total of 6 527 854 patients were enrolled in the linked datasets (1 January 1998 to 31 May 2016). We excluded patients whose AF diagnosis occurred before the study start date, those younger than 18 years at the time of AF diagnosis, or those with invalid start dates. The final eligible population consisted of 5 432 491 patients. Of these, 198 995 were newly diagnosed with AF in primary or secondary care during the study period. From this group, we excluded 35 406 individuals with a history of cancer. Of the remaining 163 549 patients, those with at least 1 year of follow-up or who experienced the cancer outcome within the first year after AF diagnosis were included in the final analytical cohort (see Supplementary material online, *Table S2*). To develop and validate the prediction model, we randomly split the dataset into derivation dataset (70%) and validation dataset (30%).

### Statistical methods

Analyses were performed using random forest imputation to impute missing values since we assumed missing data were missing at random. The outcomes included four types of cancer (lung, breast, prostate, and colorectal cancer) and all types of cancer by sex. Analyses of breast cancer were limited to women, and analyses of prostate cancer to men. To predict incident cancer within 1 year after AF diagnosis, we developed a multivariable logistic regression model using derivation dataset. When standard logistic regression showed numerical instability or divergence of estimates due to data separation, we applied ridge regression by introducing a penalty term to stabilize the estimates. The optimal penalty parameter (*λ*) was determined using 10-fold cross-validation, selecting the value corresponding to the minimum cross-validated error. While model coefficients were estimated in the derivation dataset, the model performance was evaluated in the validation dataset using the area under the receiver operating characteristic curve (AUC) with 95% confidence intervals (CI), calibration curves, decision curve analysis, and sensitivity, specificity, positive predictive value (PPV), and negative predictive values (NPV). We derived 95% CIs for the coefficients using a non-parametric bootstrap with 1000 resamples with replacement. We stratified patients by predicted risk into quintiles, as well as the top 10% and 5% risk percentiles.

As a sensitivity analysis, we used complete-case data, excluding individuals with missing values.

All statistical analyses were performed using R (version 4.4.1). We followed the Transparent Reporting of a multivariable prognostic model for Individual Prognosis or Diagnosis (TRIPOD) recommendations.^24^

### Ethical approval

The protocol was approved by the Independent Scientific Advisory Committee of the Medicines and Healthcare products Regulatory Agency (The Protocol 17_205R).

## Results

Baseline characteristics of the 163 549 patients with new-onset AF diagnosis and no prior cancer history (mean age: 75.1 ± 12.7 years; 50.0% women; 97.3% White), are presented in *Table 1*. Compared with men, women were older (78.4 vs. 71.8 years), had a lower prevalence of smoking (59.1% vs. 73.4%), and excessive alcohol intake (1.5% vs. 4.7%). Regarding comorbidities, women had higher rates of hypertension (60.3% vs. 51.7%), venous thromboembolism (6.5% vs. 5.1%), chronic kidney disease (CKD) (8.8% vs. 5.3%), anaemia (43.9% vs. 36.8%), and obesity (9.2% vs. 8.1%), while lower rates of diabetes (12.1% vs. 15.2%) and peripheral arterial disease (PAD) (5.3% vs. 8.8%) compared with men.

**Table 1:** Baseline characteristics of the study population

Characteristic	Overall	Men	Women
	*N* = 163 549^a^	*N* = 81 847^a^	*N* = 81 702^a^
**Demographics**			
Age	75.1 (12.7)	71.8 (12.9)	78.4 (11.6)
Ethnicity			
Non-White	3940 (2.7%)	2080 (2.8%)	1860 (2.5%)
White	144 462 (97.3%)	71 753 (97.2%)	72 709 (97.5%)
Missing	15 147	8014	7133
Deprivation			
Intermediate deprived quantile	99 457 (60.8%)	49 647 (60.7%)	49 810 (61.0%)
Least deprived quantile	26 356 (16.1%)	13 429 (16.4%)	12 927 (15.8%)
Most deprived quantile	37 736 (23.1%)	18 771 (22.9%)	18 965 (23.2%)
Smoking			
Never smoker	50 864 (33.6%)	20 314 (26.6%)	30 550 (40.9%)
Smoker	100 318 (66.4%)	56 161 (73.4%)	44 157 (59.1%)
Missing	12 367	5372	6995
Excessive alcohol intake	5010 (3.1%)	3810 (4.7%)	1200 (1.5%)
Family history of cancer	2037 (1.2%)	1024 (1.3%)	1013 (1.2%)
**Comorbidities**			
Heart failure	22 617 (13.8%)	11 182 (13.7%)	11 435 (14.0%)
Hypertension	91 596 (56.0%)	42 343 (51.7%)	49 253 (60.3%)
Diabetes	22 307 (13.6%)	12 444 (15.2%)	9863 (12.1%)
Stroke/SE	15 394 (9.4%)	7573 (9.3%)	7821 (9.6%)
VTE	9521 (5.8%)	4201 (5.1%)	5320 (6.5%)
PAD	11 110 (6.8%)	6509 (8.0%)	4601 (5.6%)
CKD	11 526 (7.0%)	4372 (5.3%)	7154 (8.8%)
Anaemia	65 933 (40.3%)	30 105 (36.8%)	35 828 (43.9%)
Obesity	14 086 (8.6%)	6596 (8.1%)	7490 (9.2%)
**Symptoms & clinical signs**			
Loss of appetite	772 (0.5%)	337 (0.4%)	435 (0.5%)
Weight loss	1702 (1.0%)	818 (1.0%)	884 (1.1%)
CRP			
Elevated (≥10 mg/L)	5906 (3.6%)	2771 (3.4%)	3135 (3.8%)
Normal (<10 mg/L)	9721 (5.9%)	4507 (5.5%)	5214 (6.4%)
No test	147 922 (90.4%)	74 569 (91.1%)	73 353 (89.8%)
Haemoptysis	558 (0.3%)	367 (0.4%)	191 (0.2%)
Rectal bleeding	1857 (1.1%)	919 (1.1%)	938 (1.1%)
Diarrhoea	5835 (3.6%)	2307 (2.8%)	3528 (4.3%)
Chang in bowel habit	874 (0.5%)	444 (0.5%)	430 (0.5%)
Abdominal pain or bloating	8656 (5.3%)	3804 (4.6%)	4852 (5.9%)
Urinary bleeding	1341 (0.8%)	972 (1.2%)	369 (0.5%)
PSA			
Elevated^b^	1387 (0.8%)	1384 (1.7%)	3 (0.0%)
Normal	6662 (4.1%)	6657 (8.1%)	5 (0.0%)
No test	155 500 (95.1%)	73 806 (90.2%)	81 694 (100.0%)
Breast pain or lump	668 (0.4%)	93 (0.1%)	575 (0.7%)

CKD, chronic kidney disease; PAD, peripheral artery disease; VAD, vascular dementia; CRP, C-reactive protein; PSA, prostate specific antigen.

^a^Mean (SD); *n* (%).

^b^Age-specific thresholds: 2.5 ng/mL for men aged <50 years, 3.5 ng/mL for 50–59 years, 4.5 ng/mL for 60–69 years, and 6.5 ng/mL for ≥70 years.

Supplementary material online, *Table S3* presents the baseline characteristics of patients with and without missing data. Compared with those without missing data, patients with missing values were older and fewer comorbidities and symptoms or clinical signs. Supplement Supplementary material online, *Table S4* showed the number of patients, events by the outcomes in the total, derivation, and validation datasets.

### Factors associated with 1-year cancer risk

**Figure 2:**
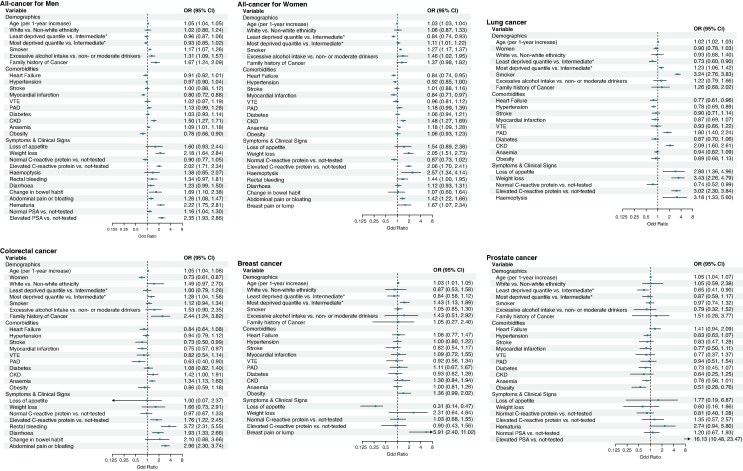
Adjusted odds ratios for 1-year cancer incidence following AF. CKD, chronic kidney disease; PAD, peripheral artery disease; VTE, venous thromboembolism. *Intermediate: individuals who were neither in the least nor in the most deprived quintile.

*Figure 2* presents adjusted odds ratios from logistic regression models assessing cancer incidence within 1 year following AF. Full results for all predictors are provided in Supplementary material online, *Table S5*. Across all cancer outcomes, older age (per 1-year increase) was associated with higher cancer risk (OR range 1.02–1.05). In the all-cancer model, shared potential risk factors in both men and women included smoking (OR 1.17 in men, 1.27 in women), excessive alcohol intake (OR 1.31 in men, 1.46 in women), family history of cancer (OR 1.67 in men, 1.37 in women), CKD (OR 1.50 in men, 1.48 in women), anaemia (OR 1.09 in men, 1.18 in women), weight loss (OR 2.18 in men, 2.05 in women), elevated CRP (OR 2.02 in men, 2.06 in women), and abdominal pain or bloating (OR 1.26 in men, 1.42 in women). In contrast, myocardial infarction was associated with lower odds (OR 0.80 in men, 0.84 in women).

Lung cancer risk was particularly elevated in individuals with weight loss (OR 3.43, 95%CI 2.26–4.79), smoking (OR 3.24, 95%CI 2.76–3.83), haemoptysis (OR 3.18, 95%CI 1.33–5.60), elevated CRP (OR 3.02, 95%CI 2.30–3.84) and loss of appetite (OR 2.88, 95%CI 1.36–4.96). The most deprived socioeconomic group, PAD, and CKD also showed increased risk. Conversely, the least deprived socioeconomic group (OR 0.73, 95% CI 0.60–0.90) and normal CRP (OR 0.74, 95% CI 0.52–0.99) were associated with lower lung cancer risk.

Colorectal cancer was associated with rectal bleeding (OR 3.72, 95%CI 2.31–5.55), abdominal pain or bloating (OR 2.96, 95%CI 2.30–3.74), family history of cancer (OR 2.44, 95%CI 1.24–3.82), diarrhoea (OR 1.93, 95%CI 1.33–2.66), elevated CRP (OR 1.76, 95%CI 1.22–2.45), most deprived socioeconomic group (OR 1.28, 95%CI 1.04–1.58), and anaemia (OR 1.34, 95%CI 1.13–1.60). Associations with reduced risk were observed for PAD (OR 0.63, 95%CI 0.40–0.90), women (OR 0.73, 95%CI 0.61–0.87), stroke (OR 0.73, 95%CI 0.50–0.99), and myocardial infarction (OR 0.75, 95%CI 0.57–0.97).

Breast cancer risk was positively associated with breast pain or lump (OR 5.91, 95%CI 2.40–11.02) and most deprived socioeconomic group (OR 1.43, 95%CI 1.13–1.89), while it was negatively associated with loss of appetite (OR 0.31, 95%CI 0.14–0.47).

For prostate cancer risk, elevated PSA was strongest association with prostate cancer (OR 16.13, 95%CI 10.48–23.47). Obesity (OR 0.51, 95%CI 0.28–0.76) and the least deprived socioeconomic group (OR 0.65, 95% CI 0.41–0.90) were associated with lower prostate cancer risk.

### Risk prediction performance

The 1-year prediction models showed moderate-to-good discrimination, with AUCs calculated using the validation dataset: AUC values were 0.69 (95%CI 0.68–0.70) for all cancer in men, 0.63 (95%CI 0.62–0.65) for all cancer in women, 0.70 (95%CI 0.68–0.73) for lung cancer (both sexes), 0.70 (95%CI 0.66–0.73) for colorectal cancer (both sexes), 0.59 (95%CI 0.53–0.65) for breast cancer in women, and 0.78 (95%CI 0.72–0.84) for prostate cancer in men. This was comparable to the discrimination in the derivation dataset, with AUC values of 0.69 (95%CI 0.68–0.70) for all cancer in men, 0.63 (95%CI 0.62–0.64) for all cancer in women, 0.70 (95%CI 0.68–0.71) for lung cancer, 0.72 (95%CI 0.70–0.74) for colorectal cancer, 0.64 (95%CI 0.60–0.68) for breast cancer in women, and 0.84 (95%CI 0.81–0.87) for prostate cancer in men.

**Figure 3:**
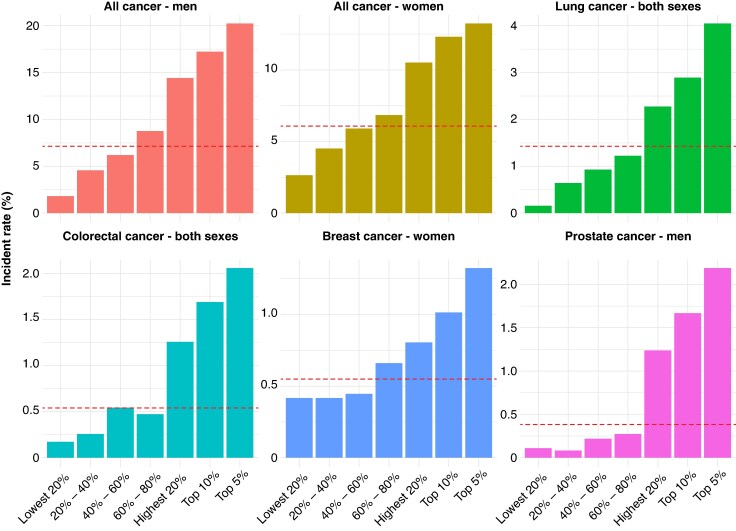
Cancer incidence rates by predicted risk level. Horizontal dashed lines indicate the overall event rate for each cancer type.

*Figure 3* shows cancer incidence rates across risk quintiles and top risk percentiles (top 10% and 5%). Higher risk groups consistently exhibited higher cancer incidence, showing a clear upward trend across the strata. The model calibration plots indicated good agreement between predicted and observed risk for all cancer types (see Supplementary material online, *Figure S1*). In these plots, the *x*-axis shows the mean predicted probability, and the *y*-axis shows the observed event rate within each decile of predicted risk. The 45° line represents perfect calibration, and the proximity of the plotted points to this line indicates that the model’s predicted probabilities were well aligned with observed outcomes. In Supplementary material online, *Figure S2*, decision curve analysis showed that the prediction model provided greater net benefit than the ‘treat-all’ or ‘treat-none’ strategies across a range of threshold probabilities. Sensitivity, specificity, PPV, and NPV are presented in Supplementary material online, *Table S5*.

### Sensitivity analysis

The result of multivariable models using imputed data was broadly consistent with the complete case analysis. No predictors with a 95% CI entirely below one in the main analysis shifted to above one in the imputed analysis (or vice versa) (see Supplementary material online, *Figure S3*). Discriminatory performance remained similar: AUC values were 0.68 (95%CI 0.66–0.70) for all cancer in men, 0.63 (95%CI 0.61–0.64) for all cancer in women, 0.71 (95%CI 0.69–0.74) for lung cancer (both sexes), 0.68 (95%CI 0.64–0.72) for colorectal cancer (both sexes), 0.56 (95%CI 0.49–0.62) for breast cancer in women, and 0.81 (95%CI 0.75–0.86) for prostate cancer in men.

## Discussion

In this nationwide study, we investigated cancer risk in patients with new-onset AF using large-scale linked UK electronic health records. Our main findings are as follows: (i) We identified potential risk factors for lung, breast, prostate, and colorectal cancer, with several overlapping predictors across cancer types; (ii) many of the identified potential risk factors for cancer are also known potential risk factors for cardiovascular disease and are modifiable, including smoking, and excessive alcohol consumption; targeting these modifiable potential risk factors may help reduce both cardiovascular disease and cancer risk in this population; and (iii) 1-year cancer prediction models based on logistic regression showed moderate-to-good predictive performance. Our models showed high NPV (>0.96) but relatively low PPV (<0.12). This shows the models clearly differentiate between patients with and without cancer, and meet the accepted NICE PPV of 3% as a threshold for fast-track investigation of suspected cancer.^23^ Identifying low or high-risk individuals may help guide decisions about cancer screening in newly diagnosed AF patients. This is consistent with the key recommendations of the recent European Society of Cardiology guidelines for the management of AF, particularly the emphasis on the AF-CARE framework and the implementation of structured, comorbidity-focused care pathways.^25^

Global cancer statistics from 2022 reported age-standardized incidence rates per 100 000 varying by region and sex.^3^ These ranged from 6.5 to 100.3 for breast cancer, 3.1 to 82.8 for prostate cancer, 2.2 to 25.1 for colon cancer, 2.0 to 16.5 for rectal cancer, and 1.5 to 51.4 for lung cancer. In the US, 2023 estimates indicated age-adjusted incidence rates per 100 000 population of 128.1 for breast cancer, 35.9 for colon and rectum, 56.3 for lung and bronchus, and 109.9 for prostate cancer.^26^ In our models, the higher-risk groups had incidence rates exceeding 1%, suggesting that our model can identify individuals at substantially elevated risk.

While several of the identified potential risk factors are consistent with findings from previous studies, we observed some discrepancies. For instance, hypertension was not associated with increased cancer incidence among AF patients, whereas prior research has reported a positive association. A pooled analysis of 12 Australian and New Zealand cohorts found hypertension to be linked to a 6–9% higher cancer risk,^27^ and a meta-analysis of 148 studies found elevated risk for specific cancers, such as kidney, breast, and colorectal cancer.^28^

Interestingly, obesity at the time of AF diagnosis was associated with lower cancer risk in our study among men, and with a non-significant trend for higher cancer risk in women. A meta-analysis of 204 studies showed strong evidence for an association between obesity and the risk of 11 types of cancer, including colon, rectal and breast cancer. In contrast, obesity was associated with a lower risk of lung cancer.^29^ A large population-based cohort study in the UK showed that body mass index was positively associated with colon cancer and post-menopausal breast cancer, and negatively associated with premenopausal breast cancer, prostate cancer, and some forms of lung cancer.^30^ Reverse causation may explain this finding, as unintentional weight loss often precedes clinical recognition of cancer.^31^ Obese individuals without cancer may have remained overweight, whereas individuals with undiagnosed cancer may have already been losing weight at the time of AF diagnosis.

Our study showed women have a lower cancer risk than men. This finding is consistent with previous studies, showing a higher prevalence of solid tumours at shared anatomic sites in men compared to women in a large US cohort.^32^

Smoking, alcohol consumption, and weight loss are well-known risk factors for cancer, which were consistently identified in our models.^3,33^ In addition, we identified other potential risk factors also known to be associated with cardiovascular conditions, such as CKD and anaemia, and cardiovascular disorders associated with cancer, such as PAD. These conditions can be prevented through appropriate control of cardiovascular risk factors. It is important to note that causality cannot be inferred for these associations. Patients with AF often have more frequent clinical encounters and diagnostic testing. This may occur even more frequently when AF patients have more comorbidities and may be driving the association of some of these common comorbidities in the AF population with higher cancer detection rates, suggesting a potential for detection bias.

Additionally, recent studies suggest that bleeding events after initiation of oral anticoagulation in AF patients may be a potential marker of undiagnosed cancer.^34,35^ A Canadian cohort study found a strong association between bleeding and subsequent cancer diagnosis (HR 4.0, 95%CI 3.8–4.3), with a significantly stronger association for cancers concordant with the bleeding site.^34^ Rectal bleeding and haemoptysis were predictors included in our models. Such findings highlight the importance of clinical vigilance in this context. Furthermore, despite advances in risk prediction, tools such as HAS-BLED remain insufficiently accurate at identifying AF patients who will not experience bleeding.^36^ Incorporating the presence, or high predicted risk, of malignant neoplasms into these models could potentially enhance their predictive performance.

Our findings must be interpreted cautiously, as Mendelian randomization studies do not support a causal link between AF and cancer development.^8^ This contrasts with heart failure, where AF appears to play a causal role.^37^ Consequently, in heart failure, treating AF or reducing its burden can reverse HF-related changes and improve survival.^38^ Nevertheless, our study's identification of reversible risk factors associated with both AF and cancer highlights the importance of early detection and integrated risk factor management to optimize outcomes within the AF Better Care Pathway.^39^

Our study focuses on the association between new-onset AF and subsequent cancer risk in the immediate period following diagnosis. However, an important emerging concept is atrial cardiomyopathy, characterized by electromechanical alterations that lead to atrial dysfunction and create a substrate conducive to AF development.^40^ Several of the potential risk factors identified in our models—such as heart failure, obesity, hypertension, stroke, alcohol consumption, smoking, and ageing—have also been linked to atrial cardiomyopathy. Although further research is warranted, targeting these factors may be relevant not only for reversing or preventing atrial cardiomyopathy, but also for reducing the risk of new-onset AF and cancer. This highlights the importance of early potential risk factor identification, timely intervention, and the promotion of healthy lifestyle behaviours. Importantly, although some of the factors identified in our risk models are potentially reversible, non-reversible causes of AF or AF-related outcomes—such as hereditary cardiomyopathies^41,42^ and channelopathies^43,44^—may also exist within the AF population and can be challenging to diagnose. Moreover, these underlying conditions may drive the development of AF even in the absence of other comorbidities.

### Limitations

A few limitations in this study warrant emphasis. First, the use of electronic health records introduces the possibility of unmeasured confounding, as some variables associated with cancer incidence may not have been captured. Also, physical inactivity and participation in endurance sports have both been associated with an increased risk of AF,^45^ while physical inactivity is also linked to cancer. Unfortunately, due to the structure of our electronic health record dataset, we were unable to reliably capture this information within our cohort. Secondly, we handled missing data using missing forest imputation under the assumption of missing at random, which allowed us to retain the full analytical sample. Missingness was not completely random, as patients with missing data tended to be older and had a lower recorded prevalence of comorbidities. However, as a sensitivity analysis, we also performed a complete-case analysis, excluding individuals with missing values for variables such as smoking and ethnicity. The results were consistent between the two approaches, supporting the robustness of our findings. Thirdly, the discrimination of some of the models was slightly lower than in the development phase. In particular, risk prediction models for all cancers in women and breast cancer in women showed relatively low AUC values, indicating that they may not be suitable for standalone use. In such cases, careful clinical assessment—including attention to cancer ‘red flags’—alongside with established screening protocols, and further risk stratification utilizing cancer prediction models may offer a more effective approach for early cancer detection. Additionally, the models are based on readily available clinical variables. Incorporating additional data, such as biomarkers (e.g. BRCA1/2 mutation status for breast cancer, or faecal immunochemical test results for colorectal cancer) and imaging findings (e.g. mammography for breast cancer), could potentially enhance model discrimination. Fourthly, while symptoms such as haematuria, haemoptysis, and abdominal pain are clinically relevant as they may reflect early manifestations of existing malignancy, they are subject to detection bias in electronic health records. Patients who visit the healthcare system less frequently or who have a higher threshold for seeking primary care may be less likely to report these symptoms. Fifth, cancer's complex pathophysiology, including age-related prevalence (e.g. bimodality, with cancers more common in both elderly and young individuals) and the dual role of some factors as both risk and protective factors for different cancer types within the same organ, alongside varying short- and long-term association strengths, are challenges for this type of methodology, and may explain the low discrimination observed for some of the models. Sixth, patients with new-onset AF were not systematically screened for cancer following their AF diagnosis. Therefore, the observed model performance reflects contemporary real-world clinical practice, with differential follow-up, where cancer diagnoses within the first year were likely driven by symptom presentation, patient help-seeking behaviour, and existing screening programs. It is possible that universal early screening in this population would have identified additional cases within the first year, potentially improving model discrimination if those diagnoses were concentrated in higher-risk quintiles. Seventh, as this was an observational study, the associations identified cannot be interpreted as causal relationships. Finally, a common concern when developing risk prediction schemes is the risk of overfitting, where the model learns the noise in the training data rather than the underlying signal. These models were internally validated, but external validation in independent cohorts with more diversified or different ethnic representation, or representing different healthcare systems, is warranted to confirm their generalizability and clinical utility (at 1 year, and for subsequent long-term follow-up after AF diagnosis), and ease the concerns about potential overfitting. As with any screening program, a cost-effectiveness evaluation is among the further steps needed before clinical implementation.

## Conclusion

We identified potential risk factors for all cancer types in men and women, lung, breast, prostate, and colorectal cancer in patients with AF. Most identified potential risk factors are also linked with cardiovascular disease. Our 1-year cancer prediction models showed moderate-to-good performance and require further validation in external cohorts.

## Supplementary Material

euaf319_Supplementary_Data

## Data Availability

Data for this study were provided by the United Kingdom’s Medicines and Healthcare Products Regulatory Agency following approval by the Independent Scientific Advisory Committee [17_205] and can be made available to other researchers following application via the CPRD website (https://www.cprd.com/).
