# Supplementary material for: Risk of cancer following presentation with new-onset atrial fibrillation using data from UK national databases
Source: Europace. 2025 Dec 11;27(12):euaf319. doi: 10.1093/europace/euaf319 (PMC12722001; doi:10.1093/europace/euaf319)

**Risk of cancer following presentation with new-onset Atrial Fibrillation**

**using data from UK National databases**

**Supplementary materials**

[Supplementary Table 1 Utilised code lists 2](#_Toc213735152)

[Supplementary Table 2 Numbers of patients excluded due to death or loss to follow-up within one year after AF diagnosis. 8](#_Toc213735153)

[Supplementary Table 2 Baseline Characteristics by missing data status 9](#_Toc213735154)

[Supplementary Table 3 Patient and Event Counts by Dataset and Outcome 11](#_Toc213735155)

[Supplementary Table 4 Adjusted odds ratios for 1-year cancer incidence following AF in impute dataset 12](#_Toc213735156)

[Supplementary Table 6 Model performance of the prediction models 18](#_Toc213735157)

[Supplementary Figure 1 Calibration plots 19](#_Toc213735581)

[Supplementary Figure 2 Decision curve analysis 20](#_Toc213735582)

[Supplementary Figure 3 Adjusted odds ratios for 1-year cancer incidence following AF in complete case analysis dataset 21](#_Toc213735583)

Supplementary Table 1 Utilised code lists

|  | **CPRD**  **READ codes** | **HES**  **ICD-10 codes** |
| --- | --- | --- |
| **Breast cancer** | - | C50.0, C50.1, C50.2, C50.3, C50.4, C50.5, C50.6, C50.8, C50.9 |
| **Colorectal cancer** | - | C18.0, C18.1, C18.2, C18.3, C18.4, C18.5, C18.6, C18.7, C18.8, C18.9, C19, C20, |
| **Lung cancer** | - | C33, C34.0, C34.1, C34.2, C34.3, C34.8, C34.9 |
| **Prostate cancer** | - | C61 |
| **Excess alcohol intake** | 136S.00, 136T.00, 136W.00, 13Y8.00, 1B1c.00, 66e..00, 66e0.00, 7P22100, 8BA8.00, 8CAv.00, 8G32.00, 8H35.00, 8H7p.00, 8HkG.00, 8HkJ.00, 9k1..00, 9k12.00, 9k1A.00, 9k1B.00, 9NN2.00, C150500, C251.11, C253.00, E01..00, E010.00, E010.11, E010.12, E011.00, E011000, E011100, E011200, E011z00, E012.00, E012.11, E012000, E013.00, E015.00, E01y.00, E01y000, E01yz00, E01z.00, E23..00, E23..11, E23..12, E230.00, E230.11, E230000, E230100, E230200, E230300, E230z00, E231.00, E231.11, E231000, E231100, E231200, E231300, E231z00, E23z.00, Eu10.00, Eu10100, Eu10200, Eu10211, Eu10212, Eu10213, Eu10300, Eu10400, Eu10411, Eu10500, Eu10511, Eu10512, Eu10513, Eu10514, Eu10600, Eu10611, Eu10700, Eu10711, Eu10712, Eu10800, Eu10y00, Eu10z00, F11x000, F11x011, F144000, F25B.00, F375.00, F394100, G555.00, G852300, J153.00, J610.00, J611.00, J612.00, J612000, J613.00, J613000, J617.00, J617000, J670800, J671000, Z191.00, Z191100, Z191200, Z191211, Z4B1.00, ZV11311, ZV57A00, ZV6D600 | E24.4, F10.1, F10.2, F10.3, F10.4, F10.5, F10.6, F10.7, F10.8, F10.9, G31.2, G62.1, G72.1, I42.6, K29.2, K70, K85.2, K86.0, Z50.2, Z71.4 |
| **Family history of Cancer** | 124H.00, 124J.00, ZV16.00, ZV16000, ZV16100, ZV16111, ZV16112, ZV16113, ZV16200, ZV16211, ZV16300, ZV16400, ZV16500, ZV16600, ZV16700, ZV16711, ZV16y00, ZV16z00, ZVu6200 | Z80 |
| **Anamia** | 1452, 1453, 1454, 1458, 4234, 4235, 145..11, 66E..00, 66E1.00, 66E2.00, 66E3.00, 66E4.00, 66E5.00, 66E6.00, 66E7.00, 66E8.00, 66EZ.00, C262000, C262100, D0...00, D0...11, D0...12, D00..00, D00..11, D00..12, D000.00, D000.11, D000.12, D001.00, D00y.00, D00y.11, D00y.12, D00y000, D00y011, D00y012, D00y100, D00yz00, D00z.00, D00z000, D00z100, D00z200, D00zz00, D01..00, D01..11, D010.00, D010.11, D010.12, D010.13, D011.00, D011.11, D011000, D011011, D011013, D011100, D011200, D011X00, D011z00, D012.00, D012.11, D012100, D012111, D012112, D012200, D012300, D012400, D012500, D012z00, D013.00, D013000, D013000, D013z00, D014.00, D014000, D014100, D014z00, D01y.00, D01y000, D01y100, D01yy00, D01yz00, D01z.00, D01z.11, D01z000, D0y..00, D0z..00, D1...00, D10..00, D100.00, D100.11, D100.12, D101.00, D101.11, D102.00, D102000, D102100, D102200, D103000, D103100, D103300, D103z00, D104211, D106.00, D106000, D106100, D106200, D106300, D106400, D106500, D106z00, D107.00, D107000, D107100, D107111, D107200, D107300, D107400, D107500, D107600, D107700, D107y00, D107z00, D10y.00, D10y000, D10yz00, D10z.00, D11..00, D110.00, D110.11, D110000, D110100, D110200, D110400, D110z00, D111.00, D111000, D111100, D111300, D111400, D111500, D111y00, D111z00, D112.00, D112000, D112011, D112012, D112100, D112200, D112z00, D112z11, D112z12, D112z13, D11z.00, D11z000, D11z100, D1y..00, D1z..00, D2...00, D20..00, D200.00, D200.13, D200.15, D200000, D200011, D200100, D200111, D200200, D200211, D200300, D200311, D200312, D200313, D200314, D200400, D200y00, D201.00, D201.11, D201000, D201100, D201111, D201200, D201211, D201311, D201400, D201412, D201500, D201600, D201611, D201612, D201700, D201800, D201z00, D201z12, D201z13, D201z14, D202.00, D203.00, D203000, D204.00, D20X.00, D20z.00, D21..00, D210.00, D210000, D210100, D210200, D210300, D210400, D210z00, D211.00, D211.11, D212.00, D212000, D213.00, D214.00, D215.00, D215000, D21y.00, D21y000, D21y011, D21y012, D21y200, D21y300, D21yy00, D21yz00, D21z.00, D21z.11, D21z.12, D21z.13, D2y..00, D2z..00, Dyu0.00, Dyu0000, Dyu0100, Dyu0200, Dyu0300, Dyu0600, Dyu1.00, Dyu1200, Dyu1300, Dyu1500, Dyu2200, Dyu2400, F11x600, F162.00, F381500, F422100, K0G..00, Q430.00, Q430000, Q430z00, Q455.00, Q455000, Q456.00, Qyu5C00, | D64, D53, D63, D62, D55, D58, D59, D57.8, D57.2, D57.1, D57.0, D51, D50, D60, D61, D52 |
| **Atrial Fibrillation** | 9Os3.00, G573000, 9Os0.00, G573400, G573100, 9Os..00, 3272, G573300, 9Os4.00, 9hF1.00, G573.00, 6A9..00, 9hF..00, 9Os2.00, 9Os1.00, 3273, 662S.00, G573500, G573z00, G573200 | I48 |
| **CKD** | Recorded eGFR values was utilised. (eGFR < 60) | N183, N184, N185, Y841, Y842, Z49 |
| **Diabetes** | C108.12, C108.13, C108011, C108012, C108211, C108212, C108411, C108412, C108511, C108512, C108711, C108712, C108811, C108812, C108911, C108912, C108A11, C108D11, C108E11, C108E12, C108F11, C108H11, C108J11, C108J12, C109.12, C109.13, C109011, C109012, C109111, C109112, C109211, C109212, C109411, C109412, C109511, C109512, C109611, C109612, C109711, C109712, C109A11, C109B11, C109C11, C109C12, C109D11, C109D12, C109E11, C109E12, C109F11, C109F12, C109G11, C109G12, C109H11, C109H12, C109J00, C109J12, C109K00, C10E.00, C10E.11, C10E000, C10E100, C10E200, C10E300, C10E311, C10E400, C10E411, C10E500, C10E511, C10E600, C10E700, C10E711, C10E800, C10E900, C10E911, C10EA00, C10EA11, C10EB00, C10EC00, C10EC11, C10ED00, C10EE00, C10EF00, C10EG00, C10EH00, C10EJ00, C10EK00, C10EL00, C10EM00, C10EM11, C10EN00, C10EN11, C10EP00, C10EP11, C10EQ00, C10F.00, C10F.11, C10F000, C10F011, C10F100, C10F200, C10F211, C10F300, C10F311, C10F400, C10F411, C10F500, C10F600, C10F611, C10F700, C10F711, C10F900, C10F911, C10FA00, C10FA11, C10FB00, C10FB11, C10FC00, C10FD00, C10FD11, C10FE00, C10FE11, C10FF00, C10FG00, C10FH00, C10FJ00, C10FJ11, C10FK00, C10FL00, C10FL11, C10FM00, C10FM11, C10FN00, C10FP00, C10FQ00, C10FR00 | E10, E11, E12, E13, E14, G590, G632, H280, H360, M142, N083, O240, O241, O242, O243 |
| **Heart failure** | 1O1..00, 388D.00, 662f.00, 662g.00, 662h.00, 662i.00, 8B29.00, G1yz100, G210100, G211100, G21z100, G232.00, G234.00, G400.00, G41z.11, G554000, G554011, G58..00, G58..11, G580.00, G580.11, G580.12, G580.13, G580.14, G580000, G580100, G580200, G580300, G580400, G581.00, G581.11, G581.13, G581000, G582.00, G584.00, G58z.00, G58z.12, G5yy900, G5yyA00, ZRad.00 | I110, I132, I130, I50 |
| **Hypertension** | 6624, 6627, 6628, 6146200, 662b.00, 662c.00, 662d.00, 662F.00, 662G.00, 662O.00, 662r.00, 7Q01.00, 8B26.00, 8BL0.00, 8I3N.00, F404200, F421300, G2...00, G2...11, G20..00, G200.00, G201.00, G202.00, G203.00, G20z.00, G20z.11, G21..00, G210.00, G210000, G210100, G211.00, G211000, G211100, G21z.00, G21z000, G21z011, G21z100, G21zz00, G22..00, G220.00, G221.00, G222.00, G22z.00, G22z.11, G23..00, G230.00, G231.00, G232.00, G233.00, G234.00, G23z.00, G24..00, G240.00, G240000, G240z00, G241.00, G241000, G241z00, G244.00, G24z.00, G24z000, G24z100, G24zz00, G2y..00, G2z..00, G672.00, G672.11, Gyu2.00, Gyu2100, L122.00, L122000, L122100, L122300, L122z00, L127.00, L127z00, L128.00, L128000, L128200, TJC7.00, TJC7z00, U60C500, U60C511, U60C51A | I10, I11, I12, I13, I15 |
| **Myocardial Infarction** | 3233, 3234, 3235, 3236, 323..00, 323Z.00, 889A.00, G30..00, G30..12, G30..13, G30..15, G30..16, G300.00, G301.00, G301000, G301100, G301z00, G302.00, G303.00, G304.00, G305.00, G306.00, G307.00, G307000, G307100, G308.00, G309.00, G30B.00, G30X.00, G30X000, G30y.00, G30y000, G30y100, G30y200, G30yz00, G30z.00, G31y100, G35..00, G350.00, G351.00, G353.00, G35X.00, G38..00, G380.00, G381.00, G384.00, G38z.00, Gyu3400 | I21, I22, I23, I241, I252 |
| **Obesity** | 1444, 7633, 7611400, 7611500, 7611600, 7613100, 7613111, 7613200, 7613300, 7613400, 7613500, 7613600, 7614100, 7615000, 7616000, 7616013, 7616015, 7616600, 7633000, 7633100, 7633200, 7642500, 212Q.00, 222A.00, 22A5.11, 22K5.00, 22K7.00, 66C..00, 66C1.00, 66C2.00, 66C4.00, 66C5.00, 66C6.00, 66CE.00, 66CZ.00, 761A500, 7633y00, 7633z00, 9OK..00, 9OK..11, 9OK1.00, 9OK2.00, 9OK3.00, 9OK4.00, 9OK5.00, 9OK6.00, 9OK7.00, 9OK8.00, 9OKA.00, 9OKZ.00, C38..00, C380.00, C380000, C380100, C380200, C380300, C380400, C380500, C380600, C380700, C38y.11, C38y000, C38y011, C38z.00, C38z000, Cyu7.00, Cyu7000, ZC2CM00, ZV45P00, ZV65319 | E66 |
| **Peripheral arterial disease** | 2G63.00, 7A10000, 7A10100, 7A10200, 7A10300, 7A12.00, 7A12000, 7A12100, 7A12111, 7A12112, 7A12300, 7A12311, 7A12312, 7A12y00, 7A12z00, 7A19200, 7A41.00, 7A41000, 7A41100, 7A41200, 7A41211, 7A41300, 7A41311, 7A41400, 7A41600, 7A41900, 7A41B00, 7A41C00, 7A41F00, 7A41y00, 7A41z00, 7A42.00, 7A42.11, 7A42000, 7A42011, 7A42012, 7A42100, 7A42111, 7A42y00, 7A42z00, 7A43.00, 7A43.11, 7A43000, 7A43011, 7A43100, 7A43111, 7A43300, 7A44000, 7A44100, 7A44300, 7A44400, 7A44y00, 7A44z00, 7A47.00, 7A47.11, 7A47.13, 7A47.14, 7A47.15, 7A47.16, 7A47000, 7A47100, 7A47200, 7A47300, 7A47400, 7A47600, 7A47700, 7A47B00, 7A47C00, 7A47D00, 7A47y00, 7A47z00, 7A48.00, 7A48.11, 7A48.12, 7A48.14, 7A48.15, 7A48.16, 7A48000, 7A48100, 7A48200, 7A48300, 7A48400, 7A48500, 7A48600, 7A48700, 7A48800, 7A48A00, 7A48B00, 7A48C00, 7A48D00, 7A48E00, 7A48y00, 7A48z00, 7A49.00, 7A49.11, 7A49.13, 7A49.14, 7A49.15, 7A49000, 7A49100, 7A49200, 7A49300, 7A49400, 7A49500, 7A49600, 7A49700, 7A49800, 7A49900, 7A49y00, 7A49z00, 7A4A.00, 7A4A.11, 7A4A.13, 7A4A.14, 7A4A000, 7A4A100, 7A4A200, 7A4A211, 7A4A212, 7A4A300, 7A4A311, 7A4A700, 7A4A800, 7A4Ay00, 7A4B000, 7A4B100, 7A4B200, 7A4B300, 7A4B400, 7A4B500, 7A4B800, 7A4B900, 7A50.00, 7A50000, 7A50100, 7A50200, 7A50300, A3A0F00, C107.00, C107000, C107100, C107300, C107400, C107z00, C108G00, C109F00, C109F11, C109F12, C10EG00, C10FF00, G700.11, G702.00, G702z00, G73..00, G73..11, G73..12, G73..13, G731.00, G731000, G731z00, G732.00, G732000, G732100, G733.00, G73y.00, G73y000, G73y100, G73y200, G73y400, G73y500, G73y511, G73y600, G73y700, G73y800, G73yz00, G73z.00, G73z000, G73z011, G73zz00, G740.12, G742400, G742500, G742600, G742700, G742900, G742z00, G74y000, G74y100, G74y200, G74y300, Gyu7400, M271.12, M271000, M271300, M271400, R054200, R054300, R055000, R055011 | I731, I738, I739, I743, I744, I745, L50, L50.1, L50.2, L50.3, L50.4, L50.5, L50.6, L50.8, L50.9, L51, L51.1, L51.2, L51.3, L51.4, L51.5, L51.6, L51.8, L51.9, L52, L52.1, L52.2, L52.8, L52.9, L53, L53.1, L53.2, L54.1, L54.2, L54.4, L54.8, L54.9, L58, L58.1, L58.2, L58.3, L58.4, L58.5, L58.6, L58.7, L58.8, L58.9, L59, L59.1, L59.2, L59.3, L59.4, L59.5, L59.6, L59.7, L59.8, L59.9, L60, L60.1, L60.2, L60.3, L60.4, L60.8, L60.9, L62, L62.1, L62.2, L62.8, L62.9, L63.1, L63.2, L63.3, L63.5, L65, L65.1, L65.2, L65.3 |
| **Systemic embolism** |  |  |
| **Stroke** | 17734, 7004300, code, Fyu5600, G60..00, G600.00, G601.00, G602.00, G603.00, G604.00, G605.00, G606.00, G60X.00, G60z.00, G61..00, G61..11, G61..12, G610.00, G611.00, G612.00, G613.00, G614.00, G615.00, G616.00, G617.00, G618.00, G619.00, G61X.00, G61X000, G61X100, G61z.00, G621.00, G622.00, G63..11, G63y000, G63y100, G64..00, G64..11, G64..12, G64..13, G640.00, G640000, G641.00, G641.11, G641000, G64z.00, G64z.11, G64z.12, G64z000, G64z100, G64z111, G64z200, G64z300, G64z400, G66..00, G66..11, G66..12, G66..13, G663.00, G664.00, G665.00, G666.00, G667.00, G668.00, G6W..00, G6X..00, Gyu6000, Gyu6100, Gyu6200, Gyu6300, Gyu6400, Gyu6E00, Gyu6F00, Gyu6G00, L440.11, L440.12 | G46.3, G46.4, G46.5, G46.6, G46.7, G46.8, I60, I61, I62.0, I63.0, I63.1, I63.2, I63.3, I63.4, I63.5, I63.8, I63.9, I64, I69.0, I69.1, I69.3, I69.4 |
| **Venous Thromboembolism** | 14A8100, 14AC.00, 7A09300, 7A09311, 7A0A100, 7A0B000, 8CMWA00, 9kg..00, 9kg0.00, 9kg0.11, 9kg1.00, 9kg2.00, F05..00, F050.00, F050000, F050100, F050300, F051.00, F051000, F051100, F051200, F051300, F051z00, F053.00, F053000, F053100, F05z.00, G401.00, G401.12, G401000, G401100, G676.00, G676000, G67A.00, G801.00, G801.11, G801.12, G801.13, G801600, G801700, G801800, G801900, G801A00, G801B00, G801C00, G801D00, G801E00, G801F00, G801G00, G801z00, G80y.11, G80y400, G80y500, G80y600, G80y700, G80y800, G81..00, G820.00, G820.11, G822.00, G822000, G823.00, G824.00, G825.00, G826.00, G827.00, Gyu8000, J420200, SP12200, ZV12800, ZV12811, ZV12900 | I26, I63.6, I67.6, I80.1, I80.2, I81, I82.0, I82.2, I82.3 |
| **Rectal bleeding** | 196B.00, 196C.00, 19E6.00, 19E6.11, 19ED.00, 19EG.00, 4762.00, 4762.11, 4793.00, 4794.00, 8HTE000, G848000, J573.00, J573.11, J573000, J573011, J573012, J573100, J573z00, J68..00, J681.11, J681.12, J681.13, J68z.00, J68z.11, J68z100, J68zz00, Q414200 |  |
| **Urinary bleeding** | 1A45.00, 1A45.11, 1A45.12, 4625.00, K0A2.00, K197.00, K197.11, K197000, K197100, K197300, K197400 |  |
| **Diarrhoea** | 19F..00, 19F..11, 19F..12, 19F2.00, 19F3.00, 19FZ.00, 19FZ.11, 19G..00, 4743.00, 4744.00, A074011, A074111, A074311, A076.11, A082.00, A082.11, A082000, A082100, A082z00, A083.00, A083.11, Ayu0H00, E264300, Eu45317, J4...13, J432.11, J433.11, J43z.11, J4z..11, J4zz.11, J521000, J525.00, Q46y100, R077100 |  |
| **Chang in bowel habit** | 19EA.00, 19EA.11, 6643.00, R078.00 |  |
| **Loss of appetite** | 1612.00, 1612.11, 1612.12, 1615.00, E275600, Eu50y12, R030.00, R030000, R030z00, ZC11.00 |  |
| **Weight loss** | 1623.00, 1625.00, 1625.11, 1627.00, 1D1A.00, 2224.00, 2287.00, 22A6.00, 22A8.00, R032.00, R034800, R2y4.00, R2y4000, R2y4z00 |  |
| **Haemoptysis** | 172..00, 172..11, 172..12, R063.00, R063000, R063100, R063z00 |  |
| **Breast pain or lump** | 1A8..00, 1A82.00, 1A83.00, 1A84.00, 1A85.00, 1A86.00, 1A8Z.00, 26B7.11, 26BD.00, 26BF.00, 26BG.00, 26BH.00, 26E..00, 26E..11, 26E2.00, 26E3.00, 26E4.00, 26E5.00, 26E6.00, 26E7.00, 26EZ.00, 26F..00, 26F1.00, 26F2.00, 26F3.00, 26F4.00, 26FZ.00, 26G..00, 26G1.00, 26G2.00, 26G3.00, 26GZ.00, 26H..00, 26H..11, 26H1.00, 26H2.00, 26HZ.00, 26I..00, 26I1.00, 26I2.00, 26I3.00, 26IZ.00, K317000, K317011, K317100, K317111, K317600 |  |
| **Abdominal pain or Bloating** | 1829.00, 196..00, 196..11, 196..12, 1962.00, 1963.00, 1968.00, 1969.00, 1969000, 196Z.00, 197..00, 197..11, 197..12, 197..13, 197..14, 1971.00, 1972.00, 1973.00, 1975.00, 1976.00, 1977.00, 1978.00, 1979.00, 197A.00, 197A.11, 197B.00, 197C.00, 197D.00, 197Z.00, 19A2.00, 19B..12, 1A53.12, 1DC5.00, 25C..00, 25C..11, 25C..12, 25C..13, 25C..14, 25C..15, 25C2.00, 25C3.00, 25C4.00, 25C5.00, 25C6.00, 25C7.00, 25C8.00, 25C9.00, 25CA.00, 25CZ.00, 25D..00, 25D..11, 25D2.00, 25D3.00, 25D4.00, 25D6.00, 25D8.00, 25D9.00, 25DA.00, 25DZ.00, 25E..00, 25EZ.00, 25F..00, 2I18.00, 2I18.12, 2I18100, E278000, R073200, R073400, R090.00, R090000, R090100, R090200, R090400, R090500, R090600, R090700, R090800, R090900, R090A00, R090C00, R090E00, R090F00, R090H00, R090J00, R090K00, R090L00, R090M00, R090N00, R090P00, R090y00, R090z00, R096.00, Ryu1000, Ryu1100 |  |

Supplementary Table 2 Numbers of patients excluded due to death or loss to follow-up within one year after AF diagnosis.

|  | <1-year follow-up, n | Death, n | Loss to follow-up, n |
| --- | --- | --- | --- |
| All cancer - men | 18929 | 11474 | 7455 |
| All cancer - women | 24379 | 15971 | 8408 |
| Lung cancer - both sexes | 46410 | 30220 | 16190 |
| Colorectal cancer - both sexes | 46980 | 30773 | 16207 |
| Breast cancer - women | 26202 | 17619 | 8583 |
| Prostate cancer - men | 20912 | 13284 | 7628 |

Supplementary Table 2 Baseline Characteristics by missing data status

| **Characteristic** | **Without missing values** | **With missing values** |
| --- | --- | --- |
|  | N = 137,983*^1^* | N = 25,566*^1^* |
| **Demographics** |  |  |
| Age | 76.0 (67.0, 83.0) | 82.0 (73.0, 88.0) |
| Women | 68,779 (49.8%) | 12,923 (50.5%) |
| Ethnicity |  |  |
| Non-White | 3,708 (2.7%) | 232 (2.2%) |
| White | 134,275 (97.3%) | 10,187 (97.8%) |
| Missing | 0 | 15,147 |
| Deprivation |  |  |
| Intermediate deprived quantile | 83,733 (60.7%) | 15,724 (61.5%) |
| Least deprived quantile | 21,953 (15.9%) | 4,403 (17.2%) |
| Most deprived quantile | 32,297 (23.4%) | 5,439 (21.3%) |
| Smoking |  |  |
| Never smoker | 46,658 (33.8%) | 4,206 (31.9%) |
| Smoker | 91,325 (66.2%) | 8,993 (68.1%) |
| Missing | 0 | 12,367 |
| Excessive alcohol intake | 4,635 (3.4%) | 375 (1.5%) |
| Family history of Cancer | 1,907 (1.4%) | 130 (0.5%) |
| **Comorbidities** |  |  |
| Heart Failure | 18,842 (13.7%) | 3,775 (14.8%) |
| Hypertension | 80,931 (58.7%) | 10,665 (41.7%) |
| Diabetes | 20,404 (14.8%) | 1,903 (7.4%) |
| Stroke/SE | 13,073 (9.5%) | 2,321 (9.1%) |
| VTE | 8,405 (6.1%) | 1,116 (4.4%) |
| PAD | 9,807 (7.1%) | 1,303 (5.1%) |
| CKD | 10,731 (7.8%) | 795 (3.1%) |
| Anaemia | 57,882 (41.9%) | 8,051 (31.5%) |
| Obesity | 13,197 (9.6%) | 889 (3.5%) |
| **Symptoms & Clinical Signs** |  |  |
| Loss of appetite | 665 (0.5%) | 107 (0.4%) |
| Weight loss | 1,462 (1.1%) | 240 (0.9%) |
| CRP |  |  |
| Elevated (>=10 mg/L) | 5,296 (3.8%) | 610 (2.4%) |
| Normal (<10 mg/L) | 8,888 (6.4%) | 833 (3.3%) |
| No test | 123,799 (89.7%) | 24,123 (94.4%) |
| Haemoptysis | 496 (0.4%) | 62 (0.2%) |
| Rectal bleeding | 1,641 (1.2%) | 216 (0.8%) |
| Diarrhoea | 5,136 (3.7%) | 699 (2.7%) |
| Chang in bowel habit | 788 (0.6%) | 86 (0.3%) |
| Abdominal pain or Bloating | 7,795 (5.6%) | 861 (3.4%) |
| Urinary bleeding | 1,182 (0.9%) | 159 (0.6%) |
| PSA |  |  |
| Elevated* | 1,177 (0.9%) | 210 (0.8%) |
| Normal | 5,972 (4.3%) | 690 (2.7%) |
| No test | 130,834 (94.8%) | 24,666 (96.5%) |
| Breast pain or lump | 615 (0.4%) | 53 (0.2%) |
| *^1^* n (%); Median (Q1, Q3) | | |

CKD = Chronic kidney disease, PAD = Peripheral artery disease**,** VAD = Vascular dementia, CRP = C-reactive protein, PSA = Prostate specific antigen

***** Age-specific thresholds: 2.5 ng/mL for men aged <50 years, 3.5 ng/mL for 50–59 years, 4.5 ng/mL for 60–69 years, and 6.5 ng/mL for ≥70 years.

Supplementary Table 3 Patient and Event Counts by Dataset and Outcome

|  | Total dataset | | Derivation dataset | | Validation dataset | |
| --- | --- | --- | --- | --- | --- | --- |
|  | Number of patients | Number of events | Number of patients | Number of events | Number of patients | Number of events |
| All cancer - men | 62918 | 4336 | 44042 | 2991 | 18876 | 1345 |
| All cancer - women | 57323 | 3503 | 40126 | 2458 | 17197 | 1045 |
| Lung cancer - both sexes | 117139 | 1163 | 81997 | 796 | 35142 | 367 |
| Colorectal cancer - both sexes | 116569 | 694 | 81598 | 505 | 34971 | 189 |
| Breast cancer - women | 55500 | 309 | 38850 | 217 | 16650 | 92 |
| Prostate cancer - men | 60935 | 221 | 42654 | 151 | 18281 | 70 |

Supplementary Table 4 Adjusted odds ratios for 1-year cancer incidence following AF in impute dataset

| **All-cancer for Men** | **OR (95%CI)** |
| --- | --- |
| Intercept | 0.00 (0.00, 0.00) |
| Demographics | |
| Age (per 1-year increase) | 1.05 (1.04, 1.05) |
| White vs Non-white ethnicity | 1.02 (0.86, 1.24) |
| Least deprived quantile vs Intermediate* | 0.96 (0.87, 1.06) |
| Most deprived quantile vs Intermediate* | 0.93 (0.85, 1.02) |
| Smoker | 1.17 (1.07, 1.26) |
| Excessive alcohol intake vs non- or moderate drinkers. | 1.31 (1.09, 1.57) |
| Family history of Cancer | 1.67 (1.24, 2.09) |
| Comorbidities | |
| Heart Failure | 0.91 (0.82, 1.01) |
| Hypertension | 0.97 (0.90, 1.04) |
| Stroke | 1.00 (0.88, 1.12) |
| Myocardial infarction | 0.80 (0.72, 0.88) |
| VTE | 1.02 (0.87, 1.19) |
| PAD | 1.13 (0.99, 1.28) |
| Diabetes | 1.03 (0.93, 1.14) |
| CKD | 1.50 (1.27, 1.71) |
| Anaemia | 1.09 (1.01, 1.18) |
| Obesity | 0.78 (0.66, 0.90) |
| Symptoms & Clinical Signs | |
| Loss of appetite | 1.60 (0.93, 2.44) |
| Weight loss | 2.18 (1.64, 2.84) |
| Normal C-reactive protein vs not-testd | 0.90 (0.77, 1.05) |
| Elevated C-reactive protein vs not-testd | 2.02 (1.71, 2.34) |
| Haemoptysis | 1.38 (0.85, 2.07) |
| Rectal bleeding | 1.34 (0.97, 1.81) |
| Diarrhoea | 1.23 (0.99, 1.50) |
| Change in bowel habit | 1.69 (1.10, 2.38) |
| Abdominal pain or bloating | 1.26 (1.08, 1.47) |
| Hematuria | 2.22 (1.75, 2.81) |
| Normal PSA vs not-testd | 1.16 (1.04, 1.30) |
| Elevated PSA vs not-testd | 2.35 (1.93, 2.86) |

| **All-cancer for Women** | **OR (95%CI)** |
| --- | --- |
| Intercept | 0.00 (0.00, 0.01) |
| Demographics | |
| Age (per 1-year increase) | 1.03 (1.03, 1.04) |
| White vs Non-white ethnicity | 1.06 (0.87, 1.33) |
| Least deprived quantile vs Intermediate* | 0.84 (0.74, 0.93) |
| Most deprived quantile vs Intermediate* | 1.11 (1.01, 1.22) |
| Smoker | 1.27 (1.17, 1.37) |
| Excessive alcohol intake vs non- or moderate drinkers. | 1.46 (1.02, 1.95) |
| Family history of Cancer | 1.37 (0.98, 1.82) |
| Comorbidities | |
| Heart Failure | 0.84 (0.74, 0.95) |
| Hypertension | 0.92 (0.85, 1.00) |
| Stroke | 1.01 (0.88, 1.16) |
| Myocardial infarction | 0.84 (0.71, 0.97) |
| VTE | 0.96 (0.81, 1.12) |
| PAD | 1.18 (0.99, 1.39) |
| Diabetes | 1.06 (0.94, 1.21) |
| CKD | 1.48 (1.27, 1.69) |
| Anaemia | 1.18 (1.09, 1.28) |
| Obesity | 1.06 (0.93, 1.23) |
| Symptoms & Clinical Signs | |
| Loss of appetite | 1.54 (0.89, 2.38) |
| Weight loss | 2.05 (1.51, 2.73) |
| Normal C-reactive protein vs not-testd | 0.87 (0.73, 1.02) |
| Elevated C-reactive protein vs not-testd | 2.06 (1.70, 2.41) |
| Haemoptysis | 2.57 (1.34, 4.14) |
| Rectal bleeding | 1.44 (1.00, 1.95) |
| Diarrhoea | 1.12 (0.93, 1.31) |
| Change in bowel habit | 1.07 (0.60, 1.64) |
| Abdominal pain or bloating | 1.42 (1.22, 1.66) |
| Breast pain or lump | 1.67 (1.07, 2.34) |

| **Lung cancer** | **OR (95%CI)** |
| --- | --- |
| Intercept | 0.00 (0.00, 0.00) |
| Demographics | |
| Age (per 1-year increase) | 1.02 (1.02, 1.03) |
| Women | 0.90 (0.78, 1.03) |
| White vs Non-white ethnicity | 0.93 (0.68, 1.40) |
| Least deprived quantile vs Intermediate* | 0.73 (0.60, 0.90) |
| Most deprived quantile vs Intermediate* | 1.23 (1.06, 1.42) |
| Smoker | 3.24 (2.76, 3.83) |
| Excessive alcohol intake vs non- or moderate drinkers. | 1.22 (0.79, 1.66) |
| Family history of Cancer | 1.26 (0.68, 2.02) |
| Comorbidities | |
| Heart Failure | 0.77 (0.61, 0.96) |
| Hypertension | 0.78 (0.69, 0.89) |
| Stroke | 0.90 (0.71, 1.14) |
| Myocardial infarction | 0.87 (0.69, 1.07) |
| VTE | 0.93 (0.66, 1.22) |
| PAD | 1.80 (1.40, 2.24) |
| Diabetes | 0.87 (0.70, 1.06) |
| CKD | 2.09 (1.60, 2.61) |
| Anaemia | 0.94 (0.82, 1.09) |
| Obesity | 0.89 (0.68, 1.13) |
| Symptoms & Clinical Signs | |
| Loss of appetite | 2.88 (1.36, 4.96) |
| Weight loss | 3.43 (2.26, 4.79) |
| Normal C-reactive protein vs not-testd | 0.74 (0.52, 0.99) |
| Elevated C-reactive protein vs not-testd | 3.02 (2.30, 3.84) |
| Haemoptysis | 3.18 (1.33, 5.60) |

| **Colorectal cancer** | OR (95%CI) |
| --- | --- |
| Intercept | 0.00 (0.00, 0.00) |
| Demographics |  |
| Age (per 1-year increase) | 1.05 (1.04, 1.06) |
| Women | 0.73 (0.61, 0.87) |
| White vs Non-white ethnicity | 1.49 (0.97, 2.70) |
| Least deprived quantile vs Intermediate* | 1.00 (0.79, 1.26) |
| Most deprived quantile vs Intermediate* | 1.28 (1.04, 1.58) |
| Smoker | 1.12 (0.94, 1.34) |
| Excessive alcohol intake vs non- or moderate drinkers. | 1.53 (0.90, 2.35) |
| Family history of Cancer | 2.44 (1.24, 3.82) |
| Comorbidities |  |
| Heart Failure | 0.84 (0.64, 1.08) |
| Hypertension | 0.94 (0.79, 1.12) |
| Stroke | 0.73 (0.50, 0.99) |
| Myocardial infarction | 0.75 (0.57, 0.97) |
| VTE | 0.82 (0.54, 1.14) |
| PAD | 0.63 (0.40, 0.90) |
| Diabetes | 1.08 (0.82, 1.40) |
| CKD | 1.42 (1.00, 1.91) |
| Anaemia | 1.34 (1.13, 1.60) |
| Obesity | 0.86 (0.59, 1.18) |
| Symptoms & Clinical Signs | |
| Loss of appetite | 1.00 (0.07, 2.37) |
| Weight loss | 1.66 (0.73, 2.91) |
| Normal C-reactive protein vs not-testd | 0.97 (0.67, 1.33) |
| Elevated C-reactive protein vs not-testd | 1.76 (1.22, 2.45) |
| Rectal bleeding | 3.72 (2.31, 5.55) |
| Diarrhoea | 1.93 (1.33, 2.66) |
| Change in bowel habit | 2.10 (0.88, 3.66) |
| Abdominal pain or bloating | 2.96 (2.30, 3.74) |

| **Breast cancer** | **OR (95%CI)** |
| --- | --- |
| Intercept | 0.00 (0.00, 0.00) |
| Demographics | |
| Age (per 1-year increase) | 1.03 (1.01, 1.05) |
| White vs Non-white ethnicity | 0.87 (0.53, 1.58) |
| Least deprived quantile vs Intermediate* | 0.84 (0.58, 1.12) |
| Most deprived quantile vs Intermediate* | 1.43 (1.13, 1.89) |
| Smoker | 1.05 (0.85, 1.30) |
| Excessive alcohol intake vs non- or moderate drinkers. | 1.43 (0.51, 2.92) |
| Family history of Cancer | 1.05 (0.27, 2.40) |
| Comorbidities | |
| Heart Failure | 1.08 (0.77, 1.47) |
| Hypertension | 1.00 (0.80, 1.22) |
| Stroke | 0.82 (0.54, 1.17) |
| Myocardial infarction | 1.09 (0.72, 1.55) |
| VTE | 0.92 (0.56, 1.34) |
| PAD | 1.11 (0.67, 1.67) |
| Diabetes | 0.93 (0.62, 1.28) |
| CKD | 1.30 (0.84, 1.94) |
| Anaemia | 1.00 (0.81, 1.26) |
| Obesity | 1.36 (0.99, 2.02) |
| Symptoms & Clinical Signs | |
| Loss of appetite | 0.31 (0.14, 0.47) |
| Weight loss | 2.31 (0.64, 4.84) |
| Normal C-reactive protein vs not-testd | 1.03 (0.68, 1.55) |
| Elevated C-reactive protein vs not-testd | 0.90 (0.43, 1.56) |
| Breast pain or lump | 5.91 (2.40, 11.02) |

| **Prostate cancer** | **OR (95%CI)** |
| --- | --- |
| Intercept | 0.00 (0.00, 0.00) |
| Demographics | |
| Age (per 1-year increase) | 1.05 (1.04, 1.07) |
| White vs Non-white ethnicity | 1.05 (0.59, 2.38) |
| Least deprived quantile vs Intermediate* | 0.65 (0.41, 0.90) |
| Most deprived quantile vs Intermediate* | 0.87 (0.59, 1.17) |
| Smoker | 0.97 (0.74, 1.32) |
| Excessive alcohol intake vs non- or moderate drinkers. | 0.79 (0.32, 1.52) |
| Family history of Cancer | 1.51 (0.28, 3.77) |
| Comorbidities | |
| Heart Failure | 1.41 (0.94, 2.09) |
| Hypertension | 0.83 (0.63, 1.07) |
| Stroke | 0.83 (0.47, 1.28) |
| Myocardial infarction | 0.77 (0.50, 1.11) |
| VTE | 0.77 (0.37, 1.37) |
| PAD | 0.94 (0.51, 1.54) |
| Diabetes | 0.73 (0.45, 1.07) |
| CKD | 0.64 (0.25, 1.25) |
| Anaemia | 0.78 (0.58, 1.01) |
| Obesity | 0.51 (0.28, 0.76) |
| Symptoms & Clinical Signs | |
| Loss of appetite | 1.77 (0.19, 6.87) |
| Weight loss | 0.60 (0.16, 1.86) |
| Normal C-reactive protein vs not-testd | 0.81 (0.40, 1.28) |
| Elevated C-reactive protein vs not-testd | 1.35 (0.57, 2.57) |
| Hematuria | 2.74 (0.94, 5.80) |
| Normal PSA vs not-testd | 1.20 (0.67, 1.93) |
| Elevated PSA vs not-testd | 16.13 (10.48, 23.47) |

Supplementary Table 6 Model performance of the prediction models

|  | Threshold | Sensitivity | Specificity | PPV | NPV |
| --- | --- | --- | --- | --- | --- |
| All cancer - men | 0.063 | 0.707 | 0.568 | 0.112 | 0.962 |
| All cancer - women | 0.054 | 0.729 | 0.457 | 0.080 | 0.963 |
| Lung cancer - both sexes | 0.012 | 0.569 | 0.724 | 0.021 | 0.994 |
| Colorectal cancer - both sexes | 0.005 | 0.709 | 0.566 | 0.009 | 0.997 |
| Breast cancer - women | 0.005 | 0.641 | 0.516 | 0.007 | 0.996 |
| Prostate cancer - men | 0.004 | 0.700 | 0.780 | 0.012 | 0.999 |

*The optimal threshold was determined using the Youden index, which maximizes the sum of sensitivity and specificity.

Supplementary Figure 1 Calibration plots: (A) All cancer -men; (B) All-cancer – women; (C) Lung cancer; (D) Colorectal cancer; (E) Breast cancer; (F) Prostate cancer


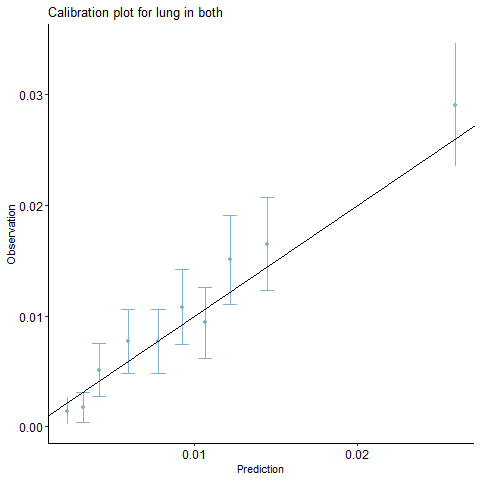

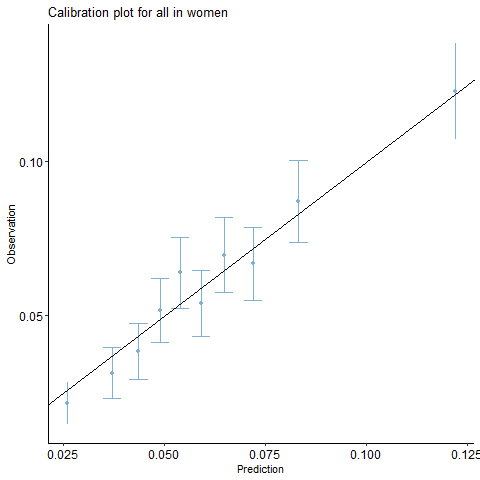

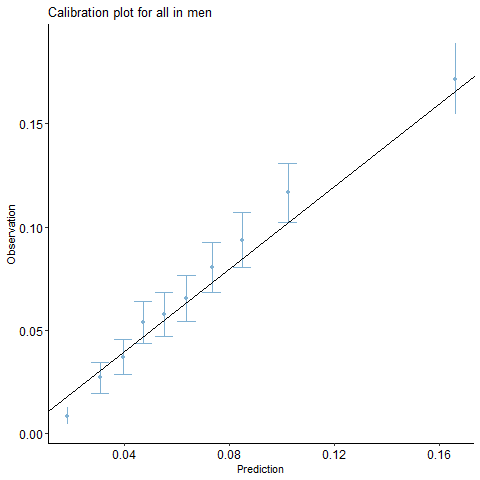
(A) (B) (C)


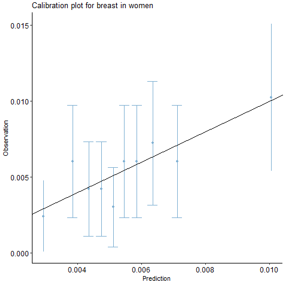

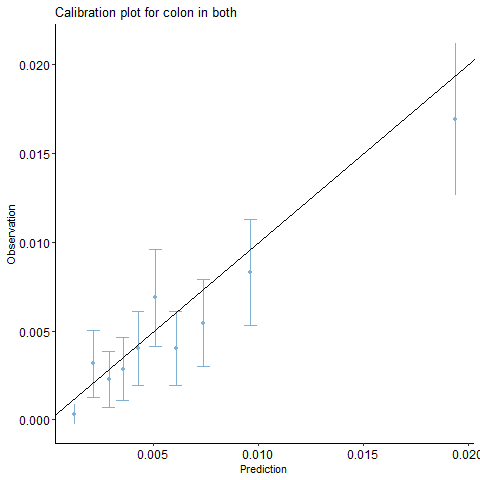
(D) (E) (F)


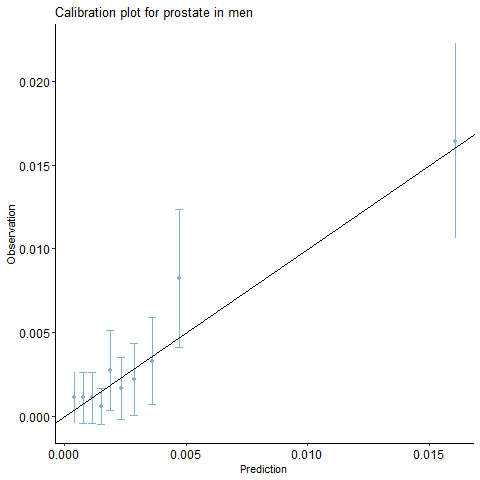


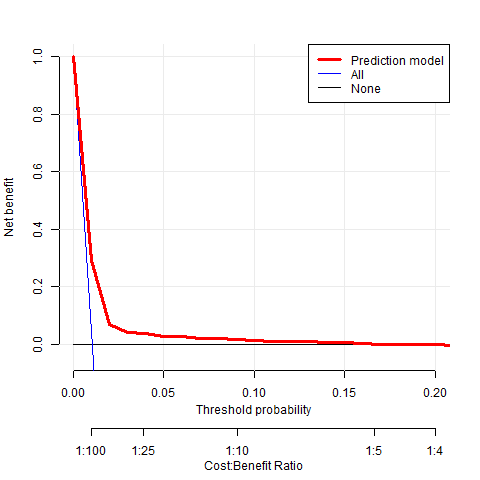
Supplementary Figure 2 Decision curve analysis: (A) All cancer -men; (B) All-cancer – women; (C) Lung cancer; (D) Colorectal cancer; (E) Breast cancer; (F) Prostate cancer


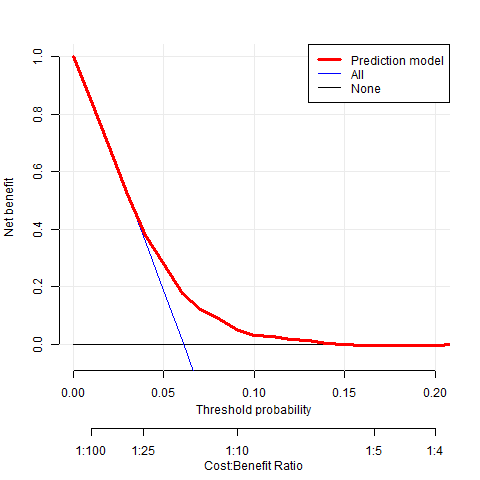

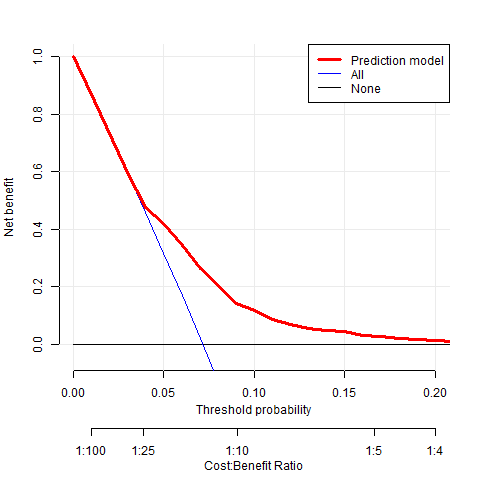
(A) (B) (C)


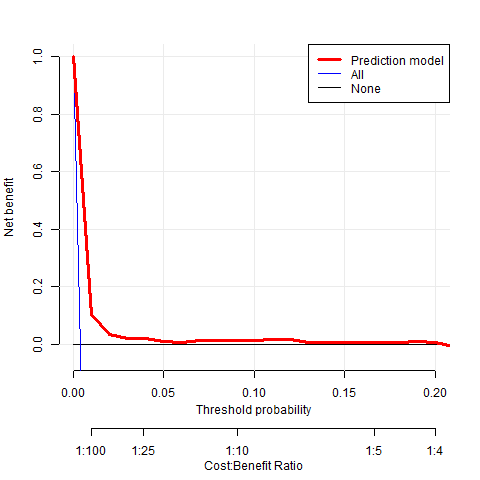

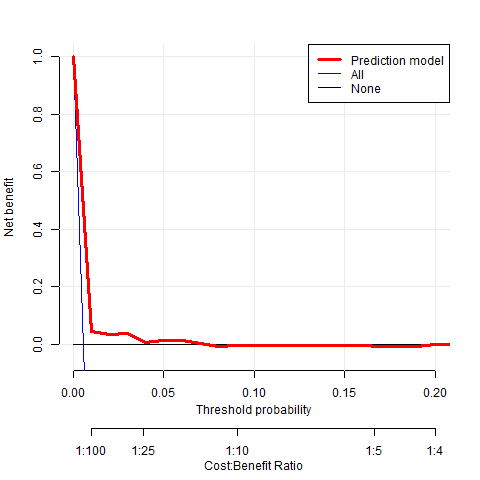

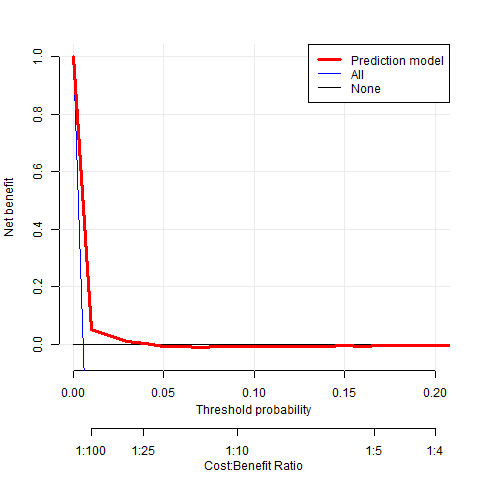


(D) (E) (F)

Supplementary Figure 3 Adjusted odds ratios for 1-year cancer incidence following AF in complete case analysis dataset


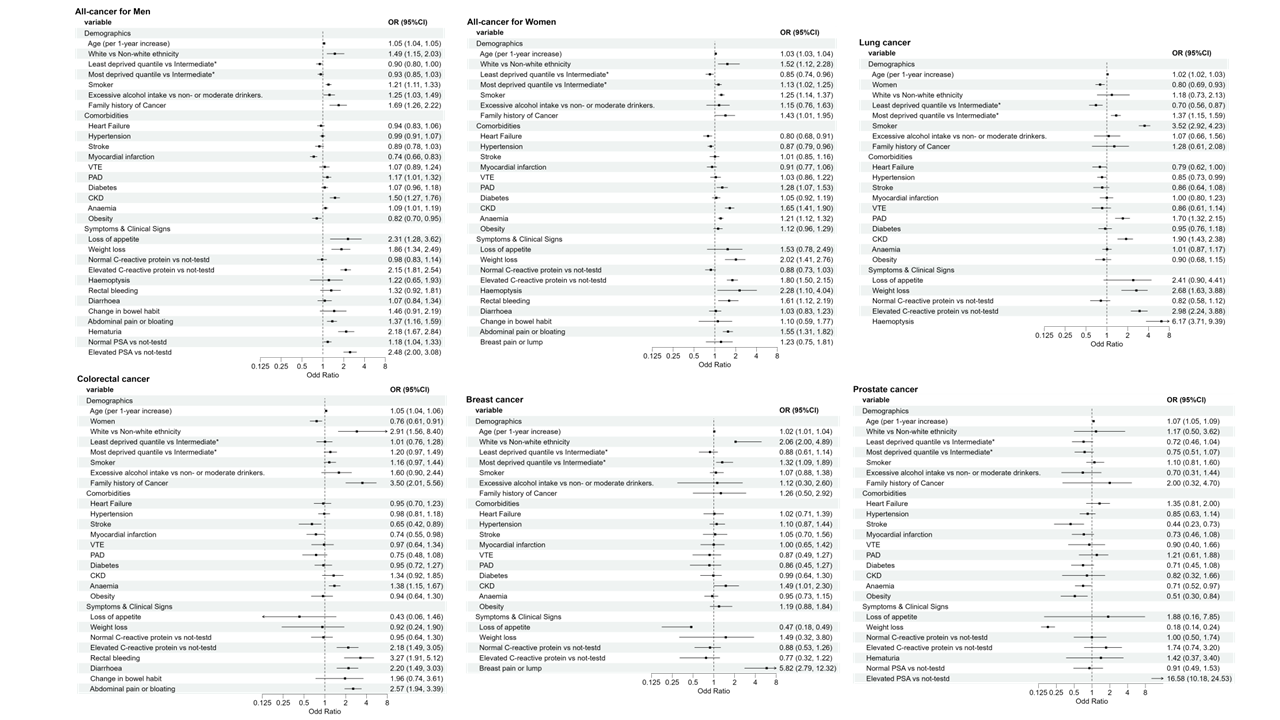

Supplement: euaf319_Supplementary_Data [file euaf319_supplementary_data.docx]
